# Breast Cancer Survival Among Males by Race, Ethnicity, Age, Geographic Region, and Stage — United States, 2007–2016

**DOI:** 10.15585/mmwr.mm6941a2

**Published:** 2020-10-16

**Authors:** Taylor D. Ellington, S. Jane Henley, Reda J. Wilson, Jacqueline W. Miller

**Affiliations:** 1 Division of Cancer Prevention and Control, National Center for Chronic Disease Prevention and Health Promotion, CDC.

Breast cancer among males in the United States is rare; approximately 2,300 new cases and 500 associated deaths were reported in 2017, accounting for approximately 1% of all breast cancers.* Risk for male breast cancer increases with increasing age (*1*), and compared with women, men receive diagnoses later in life and often at a later stage of disease (*1*). Gradual improvement in breast cancer survival from 1976–1985 to 1996–2005 has been more evident for women than for men (*1*). Studies examining survival differences among female breast cancer patients observed that non-Hispanic White (White) females had a higher survival than non-Hispanic Black (Black) females (*2*), but because of the rarity of breast cancer among males, few studies have examined survival differences by race or other factors such as age, stage, and geographic region. CDC’s National Program of Cancer Registries (NPCR)^†^ data were used to examine relative survival of males with breast cancer diagnosed during 2007–2016 by race/ethnicity, age group, stage at diagnosis, and U.S. Census region. Among males who received a diagnosis of breast cancer during 2007–2016, 1-year relative survival was 96.1%, and 5-year relative survival was 84.7%. Among characteristics examined, relative survival varied most by stage at diagnosis: the 5-year relative survival for males was higher for cancers diagnosed at localized stage (98.7%) than for those diagnosed at distant stage (25.9%). Evaluation of 1-year and 5-year relative survival among males with breast cancer might help guide health care decisions regarding early detection of male breast cancer and establishing programs to support men at high risk for breast cancer and male breast cancer survivors.

Data on survival patterns of breast cancer (*International Classification of Diseases for Oncology, Third Edition,* C50.0–C50.9)^§^ reported during 2007–2016, the most recently available data, were obtained from NPCR and restricted to those occurring in males. The data set, which covers 94% of the U.S. population, includes 45 population-based cancer registries that met U.S. Cancer Statistics (USCS) publication criteria and conducted active follow-up or linkage with CDC’s National Center for Health Statistics National Death Index (*3*). Cases with histology codes 9050–9055 (mesothelial neoplasms), 9140 (Kaposi sarcoma), and 9590–9992 (lymphomas and hematopoietic neoplasms) were excluded from analysis. The 1-year and 5-year relative survival were defined as the percentages of persons who did not die from breast cancer ≥1 year and ≥5 years after cancer diagnosis. The 1-year and 5-year relative survival for males with breast cancer diagnosed during 2007–2016 with follow-up through 2016 were calculated using the Ederer II actuarial method with the complete analysis approach to account for shorter follow-up time of cancers diagnosed in more recent diagnosis years (*4*). Using the Surveillance, Epidemiology, and End Results (SEER) statistical program (version 8.3.6; National Cancer Institute), relative survival was calculated for males with diagnosed breast cancer by race/ethnicity (four mutually exclusive groups including White, Black, Hispanic, and other [non-Hispanic Asian/Pacific Islander and non-Hispanic American/Indian Alaskan Native]), age group (<50, 50–59, 60–69, 70–79, and ≥80 years), U.S. Census region, and stage at diagnosis (SEER Summary Stage 2000^¶^ was used to characterize cancers as localized, regional, distant, or unknown stage using clinical and pathologic tumor characteristics). To allow for informal comparisons, without specifying a referent group, 95% confidence intervals for survival estimates are presented. 

Among males with breast cancer diagnosed during 2007–2016, the 1-year and 5-year relative survival was 96.1% and 84.7%, respectively (Table). One-year relative survival was 97.0% among Hispanics, 96.4% among Whites, 95.3% among other racial/ethnic groups, and 93.7% among Blacks (Figure 1). Relative survival from 1 to 5 years decreased 15.4 percentage points among Blacks (93.7% to 77.6%), 14.5 among Hispanics (97.0% to 82.5%), 10.4 among Whites (96.4% to 86.0%), and 9.1 among other racial/ethnic groups (95.3% to 86.2%).

**TABLE:** Relative survival 1 and 5 years after breast cancer diagnosis among males, by selected characteristics — United States, 2007–2016*

Characteristic	No.	Relative survival (95% CI)	
		1-year	5-year
Overall	14,805	96.1 (95.6–96.5)	84.7 (83.7–85.7)
Race/Ethnicity^†^			
White, non-Hispanic	11,306	96.4 (95.9–96.9)	86.0 (84.8–87.1)
Black, non-Hispanic	2,095	93.7 (92.3–94.9)	77.6 (74.6–80.3)
Hispanic	889	97.0 (95.1–98.2)	82.5 (78.0–86.1)
Other	392	95.3 (92.0–97.2)	86.2 (79.6–90.7)
Age group (yrs)			
<50	1,626	96.9 (95.8–97.6)	83.6 (81.2–85.7)
50–59	2,990	96.5 (95.6–97.1)	83.9 (82.0–85.6)
60–69	4,583	96.1 (95.3–96.7)	85.1 (83.4–86.6)
70–79	3,471	96.3 (95.2–97.1)	85.9 (83.3–88.1)
≥80	2,135	94.8 (92.7–96.3)	84.5 (78.8–88.7)
Census region^§^			
Northeast	3,087	95.8 (94.7–96.7)	85.9 (83.5–88.0)
Midwest	2,844	95.6 (94.4–96.5)	82.7 (80.1–85.0)
South	5,842	96.0 (95.2–96.6)	83.9 (82.2–85.5)
West	2,833	97.4 (96.3–98.1)	87.0 (84.4–89.1)
Stage at diagnosis^¶^			
Localized	6,779	99.7 (98.9–99.9)	98.7 (96.5–99.5)
Regional	6,205	98.7 (98.1–99.2)	83.7 (82.0–85.2)
Distant	1,290	70.5 (67.8–73.1)	25.9 (22.7–29.3)
Unknown	531	80.5 (76.4–84.0)	62.1 (55.7–67.8)

* Data were compiled from 45 population-based cancer registries that participate in the National Program of Cancer registries, meet the data-quality standards for inclusion in U.S. Cancer Statistics, and meet the criteria for inclusion in the survival data set, which covers approximately 94% of the U.S. population.

^†^ Racial and ethnic groups are mutually exclusive. Hispanic persons can be any race. The “other” race group contains non-Hispanic Asian/Pacific Islander and non-Hispanic American Indian/Alaska Native cases.

^§^ *Northeast*: Maine, Massachusetts, New Hampshire, New Jersey, New York, Pennsylvania, Rhode Island, and Vermont; *Midwest*: Illinois, Michigan, Minnesota, Missouri, Nebraska, North Dakota, Ohio, South Dakota, and Wisconsin; *South*: Alabama, Arkansas, Delaware, District of Columbia, Florida, Georgia, Kentucky, Louisiana, Maryland, Mississippi, North Carolina, Oklahoma, South Carolina, Tennessee, Texas, Virginia, and West Virginia; *West*: Alaska, Arizona, California, Colorado, Idaho, Montana, Nevada, Oregon, Utah, Washington, and Wyoming.

**^¶^** Surveillance, Epidemiology, and End Results Summary Stage 2000 was used to characterize cancers as localized, regional, distant, or unknown stage using clinical and pathologic tumor characteristics.

**FIGURE 1:**
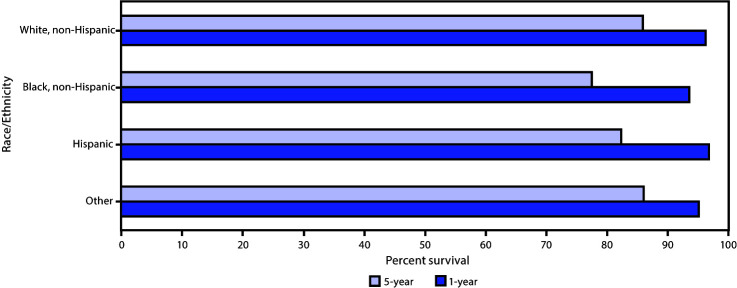
Relative 1-year and 5-year survival of male breast cancer patients, by race/ethnicity*— United States, 2007–2016^†^ * Racial and ethnic groups are mutually exclusive. Hispanic persons can be any race. The “other” race group contains non-Hispanic Asian/Pacific Islander and non-Hispanic American Indian/Alaska Native patients. ^†^ Data were compiled from 45 population-based cancer registries that participate in the National Program of Cancer registries, meet the data quality standards for inclusion in U.S. Cancer Statistics, and meet the criteria for inclusion in the survival data set, which covers approximately 94% of the U.S. population.

Approximately one third of cases were diagnosed in males aged <60 years, one third in men aged 60–69 years, and one third in men aged ≥70 years. The 1-year survival was similar for all age groups, and 5-year survival was similar for all age groups, but 1-year and 5-year differed. Survival estimates by U.S. Census region were similar; 1-year survival was 97.4% in the West, 96.0% in the South, 95.8% in the Northeast, and 95.6% in the Midwest, whereas 5-year survival was 87.0% in the West, 85.9% in the Northeast, 83.9% in the South, and 82.7% in the Midwest.

A large proportion of cases in males were diagnosed at localized (45.8%) and regional (41.9%) stages, but approximately 8.7% were diagnosed at a distant stage and 3.6% at an unknown stage. The 1-year survival was similar among males with cancer diagnosed at localized (approximately 99.7%) and regional stages (98.7%), but 5-year survival was lower among those whose cancers were diagnosed at a regional stage (83.7%) than among those diagnosed at a localized stage (98.7%). Relative survival was lowest among males with cancer diagnosed at a distant stage (1-year = 70.5%; 5-year = 25.9%) (Figure 2). Among males with breast cancer diagnosed at an unknown stage 1-year relative survival was 80.5% and 5-year was 62.1%. Although survival estimates were similar by race/ethnicity, a larger proportion of cases in Black males was diagnosed at distant stage (12.2%) than were those in males in other racial/ethnic groups (7.1% of Hispanic males, 8.1% of White males, and 10.2% of other race/ethnicity groups).

**FIGURE 2:**
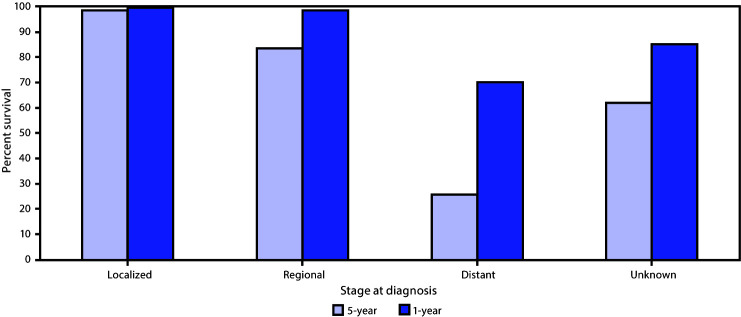
Male breast cancer relative 1-year and 5-year survival by stage at diagnosis*— United States, 2007–2016^†^ * Surveillance, Epidemiology, and End Results Summary Stage 2000 (https://seer.cancer.gov/tools/ssm/) was used to characterize cancers as localized, regional, distant, or unknown stage using clinical and pathologic tumor characteristics. ^†^ Data were compiled from 45 population-based cancer registries that participate in the National Program of Cancer registries, meet the data quality standards for inclusion in U.S. Cancer Statistics, and meet the criteria for inclusion in the survival data set, which covers approximately 94% of the U.S. population.

## Discussion

During 2007–2016, differences were observed in 1-year and 5-year relative survival among males with diagnosed breast cancer. This report found that males with breast cancer diagnosed at localized and regional stages had higher relative survival than did those whose cancers were diagnosed at a distant or unknown stage.

Results from this study show that relative survival 1 year after breast cancer diagnosis was lower among Black males than it was among White and Hispanic males. Previous studies found no significant difference between racial groups regarding receipt of primary cancer-directed treatment when stratified by stage of disease (*5*). However, differences in survival have been observed by type of treatment. The 5-year overall survival among males with breast cancer was worse for those who did not receive any treatment or who received primary radiation therapy than it was for those who received any type of mastectomy (*5*). Assuring access to optimal treatment might reduce the observed differences in relative survival by race/ethnicity.

Approximately one half of males with breast cancer received a diagnosis after it had already spread (i.e., regional or distant stage), when 5-year relative survival was lower than when diagnosed at a localized stage. It is critical that men notice any breast masses and related symptoms and seek immediate medical attention. Breast cancer symptoms among males are similar to those among females and include a painless lump or thickening in breast tissue; skin dimpling, puckering, thickening, redness, or scaling; and nipple discharge, ulceration, or retraction (*6*). Transgender females have a higher risk for breast cancer than do cisgender males, but transgender males have a lower risk than cisgender females (*7*); being aware of transgender status might help health care providers assess breast cancer risk and refer to appropriate risk-adapted early detection protocols.

Routinely discussing family health history with patients might help health care providers identify men who could be at increased risk and should undergo counseling and testing for genetic mutations.** The U.S. Surgeon General’s “My Family Health Portrait” tool can be used to collect family health history of breast, ovarian, and other cancers.^††^ Men with BRCA1 and BRCA2 mutations are more likely than are those who do not have these mutations to develop breast cancer.^§§^ If a man has a BRCA1 or BRCA2 mutation, breast self-exam training and education and yearly clinical breast exams starting at age 35 years might be recommended. Men with BRCA mutations are also at increased risk for prostate and pancreatic cancers.^¶¶^

For males who have had a breast cancer diagnosis, the risk for recurrence continues through 15 years after primary treatment and beyond (*8*). The American Society of Clinical Oncology (ASCO) recommends that male patients with breast cancer be offered genetic counseling and genetic testing for germline mutations (*8*). Continuity of care for all patients with breast cancer is recommended by ASCO and should be performed by a physician experienced in the care of patients with cancer and in breast examination, including the examination of irradiated breasts (*9*). CDC supports the National Comprehensive Cancer Control Program, which assists community programs to address the needs of cancer survivors and their caregivers_._***

The findings in this report are subject to at least two limitations. First, analyses of relative survival should be carefully interpreted. Higher relative survival among racial/ethnic groups and stage at diagnosis presented in this study might not equate to a lower mortality rate (*10*). Second, analyses based on race and ethnicity might be biased if race and ethnicity were systematically misclassified; ongoing efforts are made to ensure that this information is as accurate as possible.^†††^

CDC’s NPCR collects information about cancers diagnosed in 46 states, the District of Columbia, Puerto Rico, the U.S. affiliated Pacific Island Jurisdictions, and the U.S. Virgin Islands, and is an important data source for rare cancers. Using high quality cancer surveillance to evaluate relative survival among males with breast cancer might help guide health care decisions regarding breast cancer testing and treatment among males and establishing programs to support men at high risk for breast cancer and male breast cancer survivors.

**Summary**

**What is already known about this topic?**

Breast cancer can occur in males; approximately 2,300 new male breast cancer diagnoses and 500 associated deaths occurred in the United States in 2017.

**What is added by this report?**

During 2007–2016, relative 1- and 5-year survival for males with diagnosed breast cancer were 96.1% and 84.7%, respectively. Five-year survival was lowest among cancers diagnosed at a distant stage (25.9%) and highest among those diagnosed at a localized stage (98.7%).

**What are the implications for public health practice?**

Using high-quality cancer surveillance data to evaluate 1-year and 5-year relative survival among males with breast cancer might help guide health care decisions regarding breast cancer testing and treatment among males and establishing programs to support survivors and men at high risk for developing breast cancer.

## Footnotes

* https://www.cdc.gov/cancer/dataviz.

† https://www.cdc.gov/cancer/npcr/index.htm.

§ http://www.iacr.com.fr/index.php?option=com_content&view=category&layout=blog&id=100&Itemid=577.

¶ https://seer.cancer.gov/tools/ssm/.

** https://www.cdc.gov/cancer/family-health-history/index.htm.

†† https://phgkb.cdc.gov/FHH/html/index.html.

§§ https://www.cdc.gov/genomics/disease/breast_ovarian_cancer/medical_options.htm.

¶¶ https://www.cdc.gov/cancer/breast/men/index.htm.

*** https://www.cdc.gov/cancer/ncccp/priorities/cancer-survivor-caregiver.htm.

††† https://www.cdc.gov/cancer/uscs/technical_notes/interpreting/race.htm.
